# Supplementary material for: Investigation of multiple-dynein transport of melanosomes by non-invasive force measurement using fluctuation unit χ
Source: Sci Rep. 2019 Mar 25;9:5099. doi: 10.1038/s41598-019-41458-w (PMC6433852; doi:10.1038/s41598-019-41458-w)
Supplement: Supplementary file 1 — Supplementary Figures [file 41598_2019_41458_MOESM1_ESM.pdf]

Supplementary Information

Supplementary Figures S1–S9

Investigation of multiple-dynein transport of melanosomes by non-invasive force measurement using fluctuation unit χ

Shin Hasegawa, Takashi Sagawa, Kazuho Ikeda, Yasushi Okada, and Kumiko Hayashi

Supplementary Figure S1

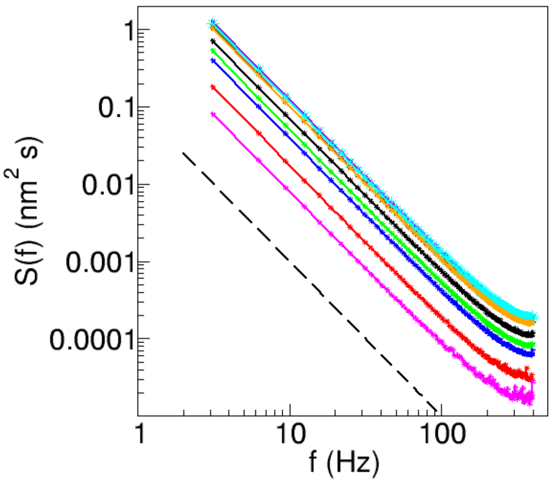

The power spectrum density (equation (11) of the main text) of the position of *X* of a melanosome for a constant velocity segment (n=10). The dotted line represents $\propto f^{-2}$.

Supplementary Figure S2

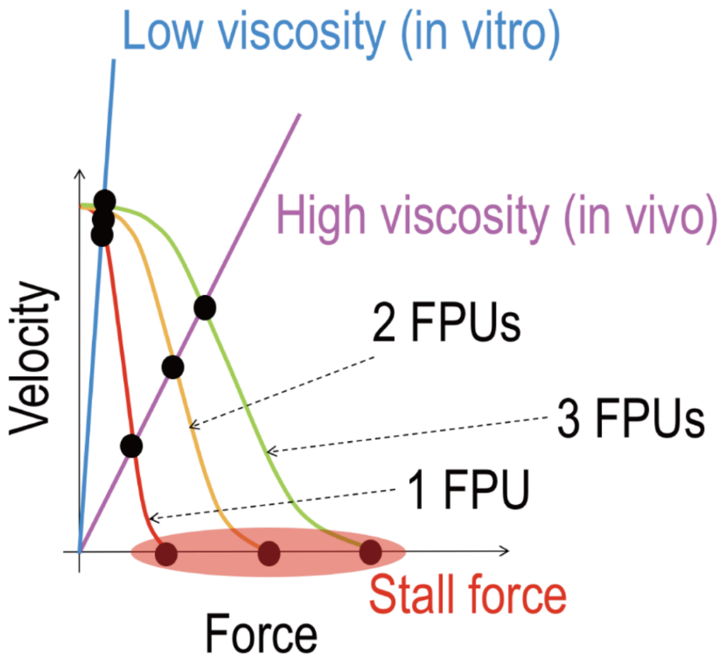

Force–velocity model of the motors considered in this study. The model was introduced in the previous study[1]. The blue and purple lines represent the relation $F = \Gamma v$ in the cases of low and high viscosities. When Γ is large, the drag forces are considered to show a quantal behavior as well as the stall forces of the motors.

Supplementary Figure S3

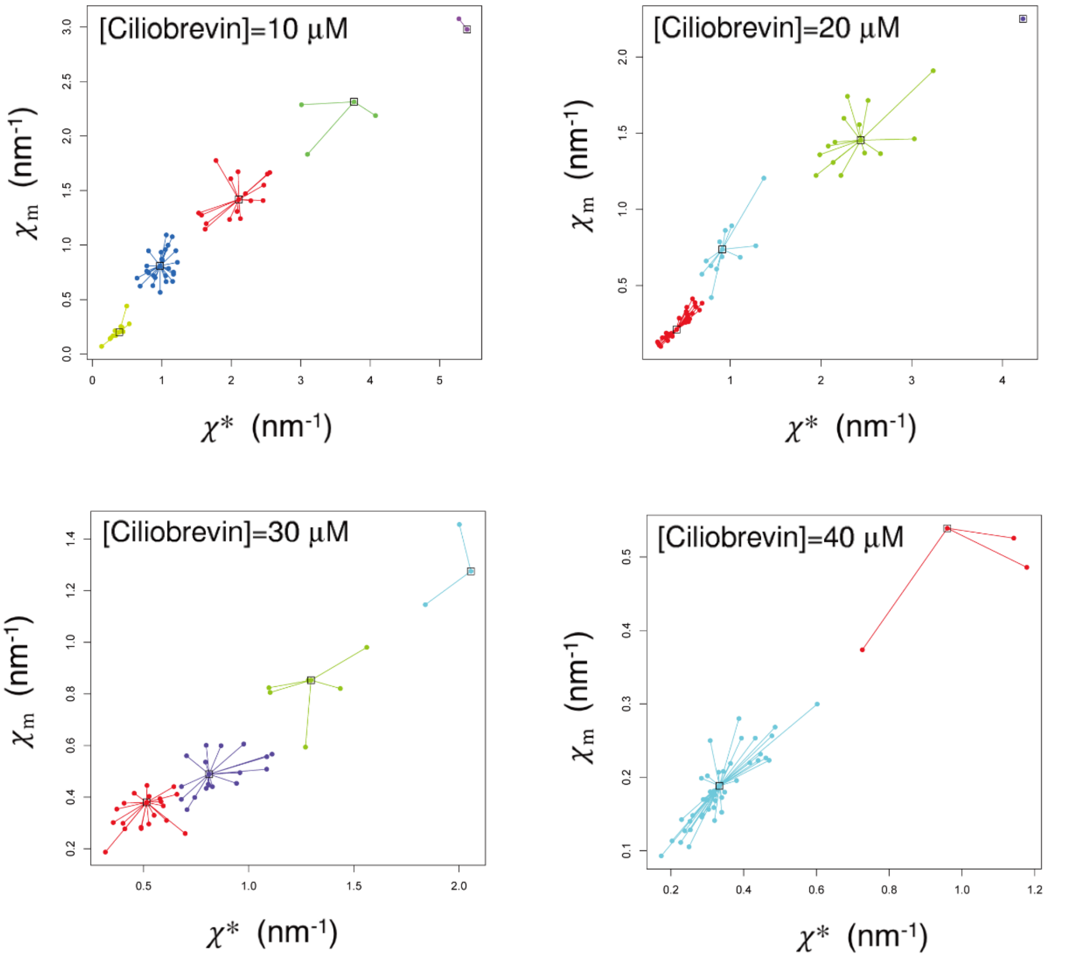

Results of the affinity propagation analysis (see Methods) with $q = 0.3$ for the data in Fig. 5. The clustering of each datum point in Fig. 5 was decided by this analysis.

Supplementary Figure S4

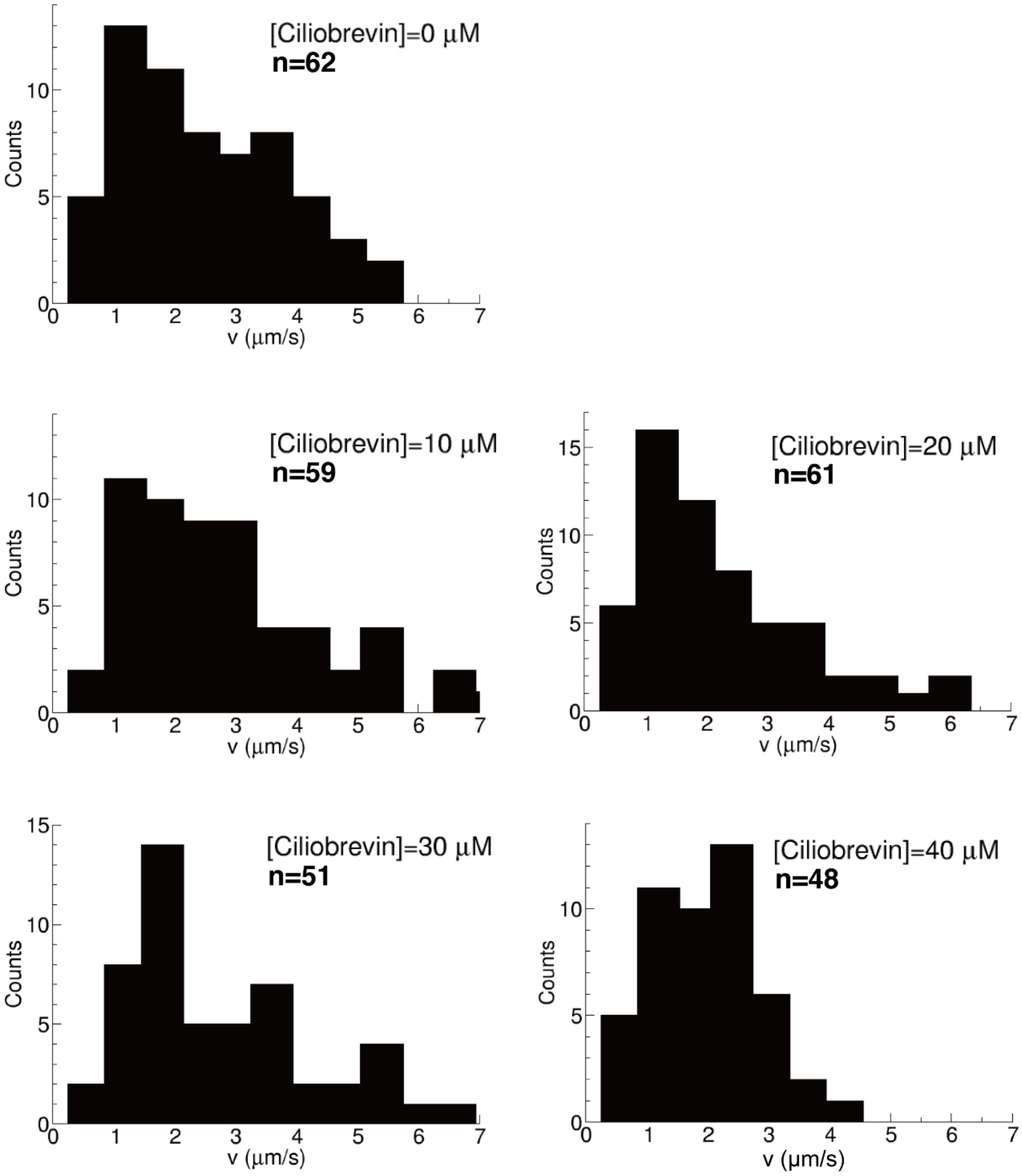

Distributions of the velocities at the constant velocity segment. The velocity distributions did not show multiple peaks clearly, unlike the drag force distributions (Fig. 4d and Fig. 5a–d).

Supplementary Figure S5

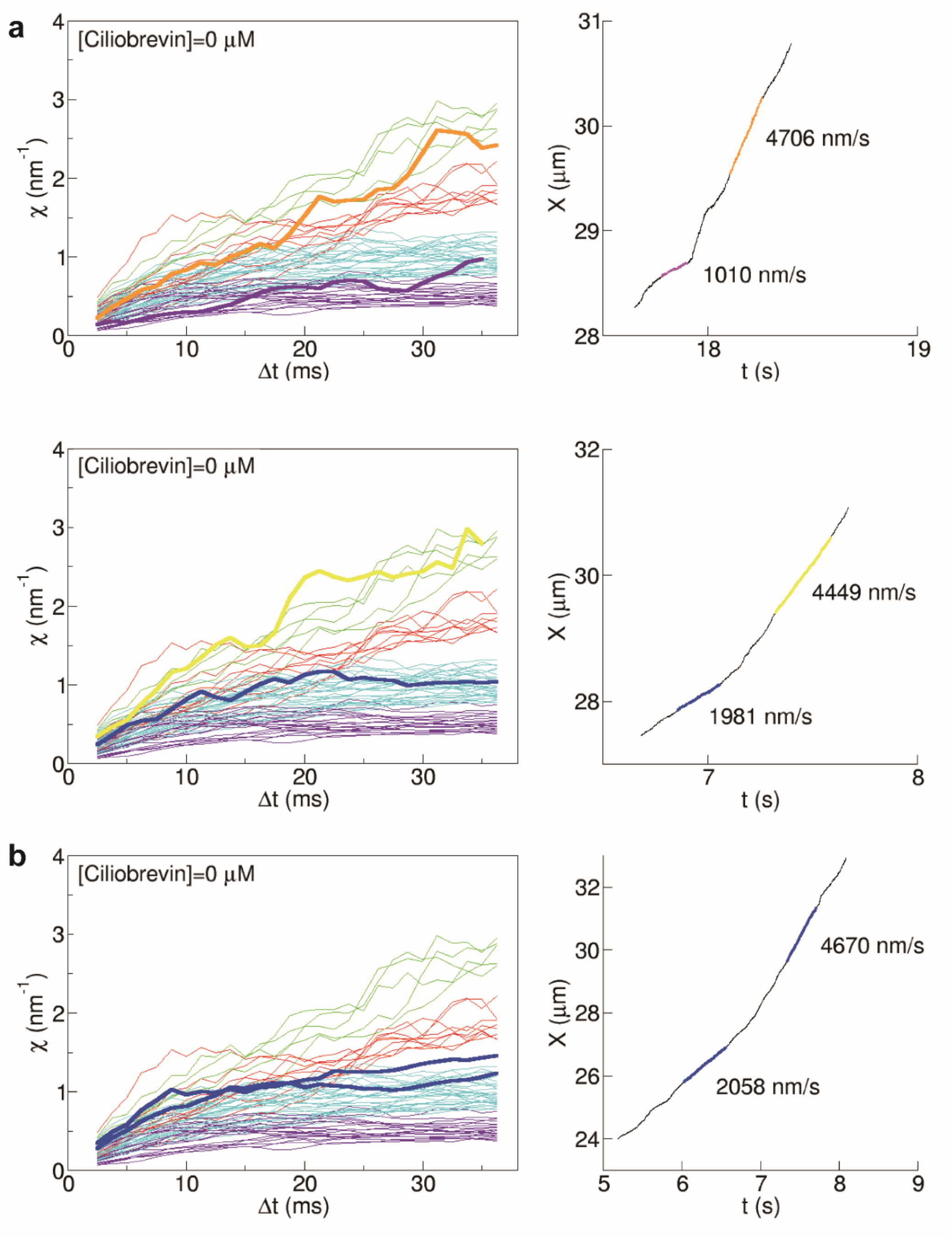

Velocity changes of the melanosomes during the aggregation process. Sometimes, velocity changes of the melanosomes were observed during the aggregation process (right panels). From the calculation of χ (left panels), we found two patterns. Here, the thick curves in the χ–Δt graphs represent χ calculated from the constant velocity segments (colored parts in the trajectories in the right-hand panels). In one case, the number of FPUs changed correspondingly to the change in velocity (**a**). In the other case, the number of FPUs did not change with the change in velocity (**b**).

Supplementary Figure S6

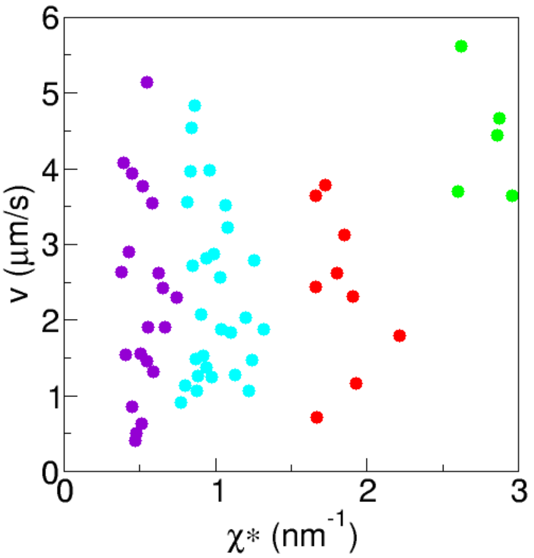

The relation between χ^* and v of the constant velocity segments in the case [ciliobrevin] = 0 μM. v as a function of χ^* investigated in Fig. 3a was plotted again for each FPU. Each color represents a cluster of Fig. 3a.

Supplementary Figure S7

a

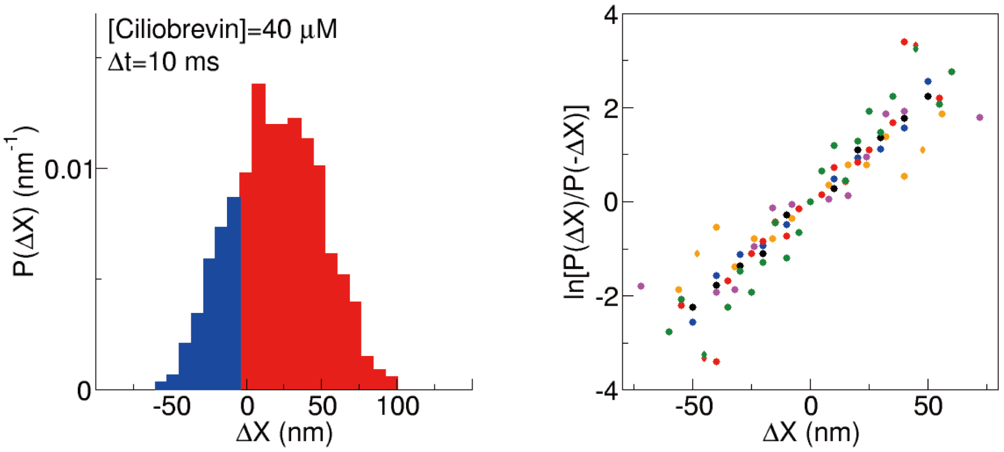

b

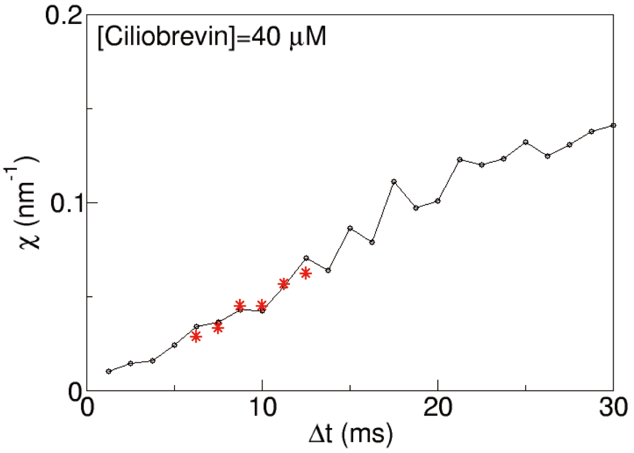

For 6.25 ms ≤ Δt < 12.5 ms in the case [ciliobrevin]=40 μM, $P(\Delta X) \neq 0$ for $\Delta X < 0$ (**a,** left panel). Then using an example time course of a melanosome in the case [ciliobrevin]=40 μM, χ_{FT} (equation (4)) was calculated as a slope of the graph in the right panel (Δt=6.25 ms (orange), 7.5 ms (pink), 8.75 ms (black), 10 ms (blue), 11.25 ms (red), 12.5 ms (green)). χ_{FT} (red symbols) is compared with χ (equation (6)) for the range of Δt (**b**).

Supplementary Figure S8

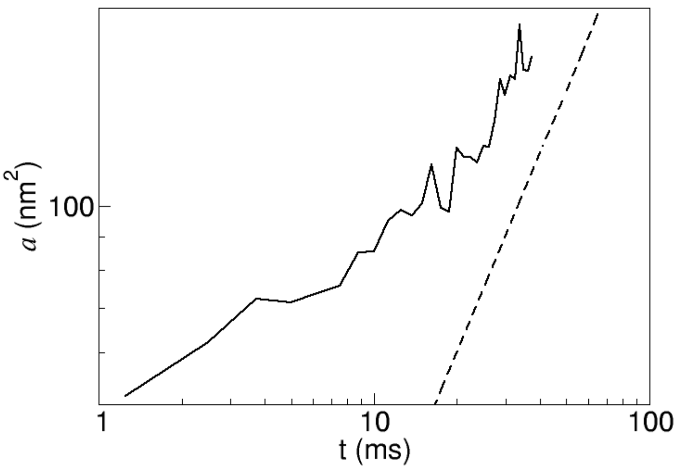

a in equation (6) as a function of Δ*t*. The dotted line represents ∝ *t*.

Supplementary Figure S9

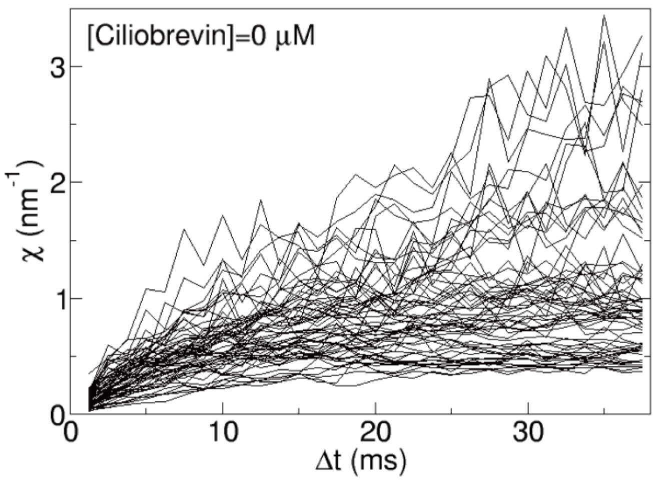

χ as a function of Δt without the smoothing filter (see Methods). Note that the graphs in Fig. 3a represent χ after the filter.

Supplementary References

1. Hayashi K, Tsuchizawa S, Iwaki M, Okada Y. Application of the fluctuation theorem for non-invasive force measurement in living neuronal axons. *Molecular Biology of the Cell* (2018) https://doi.org/10.1091/mbc.E18-01-0022
